# Risk Factors and Development of a Predictive Model for In-Hospital Mortality in Hemodynamically Stable Older Adults with Urinary Tract Infection

**DOI:** 10.3390/medicina61091625

**Published:** 2025-09-08

**Authors:** Tzu-Heng Cheng, Wei Lu, Chen-Bin Chen, Chen-June Seak, Chieh-Ching Yen

**Affiliations:** 1 Department of Emergency Medicine New Taipei Municipal Tucheng Hospital, New Taipei City 236, Taiwan; b9502086@cgmh.org.tw (T.-H.C.); roi.lu.1011@gmail.com (W.L.); ans76ers@gmail.com (C.-B.C.); 2 College of Medicine, Chang Gung University, Taoyuan 333, Taiwan; 3 Department of Emergency Medicine, Chang Gung Memorial Hospital, Linkou Branch, Taoyuan 333, Taiwan

**Keywords:** urinary tract infection, older patient, predictive model, emergency department

## Abstract

*Background and Objectives*: Urinary tract infections (UTIs) are a major cause of emergency department (ED) visits and hospital admissions among older adults. Although most seniors present hemodynamically stable, a sizeable fraction deteriorate during hospitalization, and no ED-specific tool exists to identify those at greatest risk. We sought to determine risk factors for in-hospital mortality in this population and to develop a predictive model. *Materials and Methods*: We analyzed the MIMIC-IV-ED database (2011–2019) and enrolled culture-confirmed UTI patients aged ≥ 65 years who were hemodynamically stable—defined as a systolic blood pressure ≥ 100 mm Hg without vasopressor support. Demographics, comorbidities, triage vital signs, and initial laboratory tests were extracted. Least Absolute Shrinkage and Selection Operator (LASSO) regression with 10-fold cross-validation was performed for variable selection. Discrimination was quantified with the C-statistic, calibration with the Hosmer–Lemeshow test, and clinical utility with decision curve analysis. Internal validation was assessed via 1000-sample bootstrap resampling. *Results*: Among 1571 eligible encounters (median age 79 years, 33% male), in-hospital mortality was 4.5%. LASSO selected eight variables; six remained significant in multivariable analysis: age, systolic blood pressure, oxygen saturation, white blood cell count, red cell distribution width, and blood urea nitrogen. The predictive nomogram demonstrated a C-statistic of 0.73 (95% CI 0.66–0.79) and outperformed traditional early warning scores. *Conclusions*: A six-variable nomogram may stratify mortality risk in hemodynamically stable older adults with UTI. Because the model was developed in a single U.S. tertiary-care ED, it remains hypothesis-generating until validated in external, multicenter cohorts to confirm generalizability.

## 1. Introduction

Urinary tract infections (UTIs) are among the most common infections worldwide and pose a significant health burden. Global incidence of UTI has been rising, with billions of cases annually and substantial associated morbidity [1]. Severe UTIs can lead to complications such as renal failure, sepsis, and even death [2,3,4]. In the emergency department (ED) setting, UTIs constitute a frequent cause of visits and hospital admissions. In the United States alone, over one million ED visits (roughly 1.8–2% of all ED visits) each year are attributed to UTIs [5]. UTIs are broadly classified as uncomplicated or complicated. Uncomplicated UTIs occur in otherwise healthy, non-pregnant women with a functionally and anatomically normal urinary tract and typically respond to short courses of oral antibiotics, whereas complicated UTIs arise when any structural or functional abnormality of the genitourinary tract—or a systemic host factor such as indwelling catheters, obstruction, nephrolithiasis, immunosuppression, male sex, or advanced age—predisposes the patient to treatment failure or severe infection [6,7]. A considerable proportion of UTI cases require inpatient care; for example, about two-thirds of ED patients presenting with complicated UTI end up hospitalized [8]. Notably, fewer than 10% of UTI-related ED visits initially meet the criteria for severe sepsis or septic shock, indicating that many patients are hemodynamically stable upon presentation despite harboring serious infection [5].

Older adults in the ED represent a vulnerable subgroup of UTI patients with heightened risks and poorer outcomes. Over 60% of ED patients with complicated UTI are aged 65 years or older [5]. Age-related factors contribute to this burden: immunosenescence, multiple chronic comorbidities, and increased exposure to healthcare environments all predispose older patients to more severe UTI manifestations [9]. UTIs in older adults are more likely to progress to complications such as bacteremia, sepsis, delirium, or acute organ dysfunction [10,11,12,13,14]. One hospital study reported this sharp age gradient in outcomes with in-hospital mortality rates of 0% in patients < 65 years, 0.3% in those 65–80, and 2.2% in those > 80 with UTI [15]. Given that UTIs frequently precipitate sepsis in seniors, it is not surprising that even initially stable older UTI patients face a significant risk of deterioration.

Timely recognition of older UTI patients at risk for clinical decline and poor outcomes is essential for guiding management decisions—including need for hospitalization, intensive monitoring, or early aggressive therapies—especially when patients are hemodynamically stable at presentation. However, studies examining prognostic factors in this ED population remain lacking. Therefore, we aim to investigate the risk factors for in-hospital mortality in hemodynamically stable older adults with UTI and to develop a predictive model.

## 2. Materials and Methods

### 2.1. Data Sources and Population

We conducted this study using the MIMIC-IV-ED database [16,17], which represents a comprehensive collection of more than 400,000 anonymized ED visits at Beth Israel Deaconess Medical Center, Boston, MA, from 2011 through 2019. Access to this publicly available dataset required the completion of a Collaborative Institutional Training Initiative certification by the corresponding author (certification No. 59843370). The public and anonymized status of the data eliminated the need for ethical review board approval. The study’s framework adhered to the Transparent Reporting of a multivariable prediction model for Individual Prognosis or Diagnosis Statement (TRIPOD) [18].

**Figure 1:**
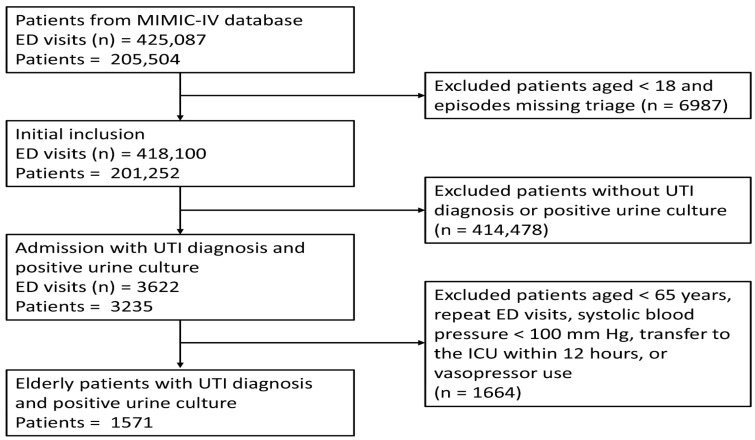
Flow diagram of patient selection. ED: emergency department; UTI: urinary tract infection; ICU: intensive care unit.

Our inclusion criteria were older adults (≥65 years) with ED-diagnosed UTI requiring hospitalization. Exclusion criteria were as follows: systolic blood pressure (SBP) under 100 mmHg at triage, vasopressor use during ED stay, ICU admission within the first 12 h, and recurrent ED visits. Hemodynamic stability was defined as absence of shock at triage. Vasopressor use was defined as norepinephrine administration or its combination with vasopressin or epinephrine. UTI identification relied on the established International Classification of Diseases, 9th and 10th Revisions (ICD-9/ICD-10) with prior validation [19]. Culture-confirmed urinary tract infections served as the definitive diagnostic standard, leading to the exclusion of cases with negative urine cultures. Figure 1 provides a flow diagram detailing our patient selection process.

### 2.2. Data Collection

Data collection encompassed demographic, clinical, and laboratory parameters obtained during the initial ED arrival. Patient demographics captured age, sex, race, and insurance coverage. Clinical assessments incorporated Emergency Severity Index (ESI), triage vital signs (systolic and diastolic blood pressure, heart rate, respiratory rate, body temperature, and oxygen saturation), and medical history including hypertension, diabetes mellitus, heart failure, cerebrovascular accident, liver disease, chronic kidney disease, malignancy, and Charlson Comorbidity Index (CCI). Initial laboratory evaluations comprised complete blood count parameters (white blood cell count [WBC], hemoglobin, red cell distribution width [RDW], platelet count), coagulation studies (prothrombin time), electrolyte panels (sodium, potassium), liver enzymes (aspartate aminotransferase [AST], alanine aminotransferase [ALT]), bilirubin levels, glucose, renal function tests (blood urea nitrogen [BUN], creatinine), urinalysis findings (leukocytes, erythrocytes), and cultured pathogen identification. Physiologically implausible vital sign and laboratory values were flagged as outliers and handled as missing data per MIMIC-EXTRACT protocols [20,21]. In-hospital mortality served as our primary outcome.

### 2.3. Model Development and Statistical Analysis

Demographic characteristics, vital signs, medical history, laboratory results, and clinical outcomes were described using frequencies and percentages for categorical variables, while continuous variables were summarized as medians with interquartile ranges. We stratified patients into survivor and non-survivor groups. Comparisons between groups were conducted using either Chi-square or Fisher’s exact tests for categorical data, and the Mann–Whitney U test for continuous data. Variables with less than 20% missing observations were included as candidates for predictive model development. Missing values were imputed using multivariate imputation by chained equations (MICE) via the mice package [22]. The extent of missing data for each variable is detailed in Supplementary Materials Figure S1.

We employed Least Absolute Shrinkage and Selection Operator (LASSO) regression combined with 10-fold cross-validation to identify risk factors associated with in-hospital mortality [23]. This approach applies an L1 penalty to the regression coefficients, effectively shrinking some coefficients to zero, which facilitates variable selection and reduces model complexity to prevent overfitting [24]. The optimal regularization parameter (λ) was chosen within minimum binomial deviance via cross-validation. Subsequently, predictors selected by LASSO with *p* value < 0.05 were incorporated into a multivariable logistic regression model to develop the final predictive model. Model discrimination was assessed using the concordance (C) statistic, while overall calibration was examined using the Hosmer–Lemeshow χ^2^ goodness-of-fit test and graphical calibration plot. Models whose Hosmer–Lemeshow *p* value > 0.05 were regarded as well-calibrated, indicating good agreement between observed and expected risks. We employed bootstrap resampling for the internal validation of our model, fitting regression models to 1000 bootstrap replicates drawn with replacement from the original sample of UTI patients to estimate optimism in model discrimination. The apparent C-statistic was subsequently adjusted for this optimism to yield a bias-corrected estimate [25]. Furthermore, clinical utility was assessed with decision curve analysis (DCA), which plots net benefit across a continuum of threshold probabilities. We compared the predictive model’s discriminative performance with that of the Modified Early Warning Score (MEWS) [26] and National Early Warning Score (NEWS) [27] using DeLong’s test. All analyses were executed in R version 4.3.2 (R Foundation for Statistical Computing, Vienna, Austria). Two-sided *p* values < 0.05 were interpreted as statistically significant. Patients were followed until hospital discharge or in-hospital death.

## 3. Results

### 3.1. Patient Characteristics

A total of 1571 hemodynamically stable older adults (median age 79 years, IQR 72–85; 33% male) were admitted with UTI between 2011 and 2019 (Figure 1). Overall in-hospital mortality was 4.5%. Most encounters involved White patients (74.7%) and those insured by Medicare (71.6%), and just over half were triaged as ESI level 2 (52.5%). During the admission, 14.4% required transfer to an ICU.

### 3.2. Clinical and Laboratory Differences Between Survivors and Non-Survivors

Differences between survivors and non-survivors are described in Table 1. Non-survivors were older (median 82 years [IQR 74–88] vs. 79 years [72,73,74,75,76,77,78,79,80,81,82,83,84,85], *p* = 0.014) and bore a heavier comorbidity burden, with hypertension (74.3% vs. 60.3%, *p* = 0.026), congestive heart failure (48.6% vs. 29.2%, *p* = 0.001), and a higher CCI (median 8 [4–11] vs. 6 [4–9], *p* = 0.002) all more prevalent or elevated in the non-survivor group. At triage, non-survivors had lower SBP (median 122 mmHg [115–138] vs. 135 mmHg [121–152], *p* < 0.001) and displayed more pronounced laboratory derangements, including higher WBC (9.9 × 10^3^ µL^−1^ [7.1–14.3] vs. 8.2 × 10^3^ µL^−1^ [6.2–11.1], *p* = 0.002), greater RDW (15.7% [14.1–18.0] vs. 14.7% [13.6–16.2], *p* < 0.001), and higher BUN (31 mg dL^−1^ [20.2–44.5] vs. 23 mg dL^−1^ [16.0–35.0], *p* = 0.001). Across the cohort, Escherichia coli was the most common urinary pathogen (37.1%), followed by *Enterococcus* spp. (15.7%), *Klebsiella* spp. (10.4%), yeast (8.6%), *Proteus* spp. (5.2%), and *Pseudomonas* spp. (4.9%). The microbiological spectrum differed between groups (*p* < 0.001). Compared with survivors, non-survivors had a lower prevalence of E. coli (21.4% vs. 37.8%) but a markedly higher prevalence of yeast (24.3% vs. 7.9%). Regarding outcomes, non-survivors required ICU transfer far more often than survivors (74.3% vs. 11.7%, *p* < 0.001).

### 3.3. Variable Selection and Model Development

**Figure 2:**
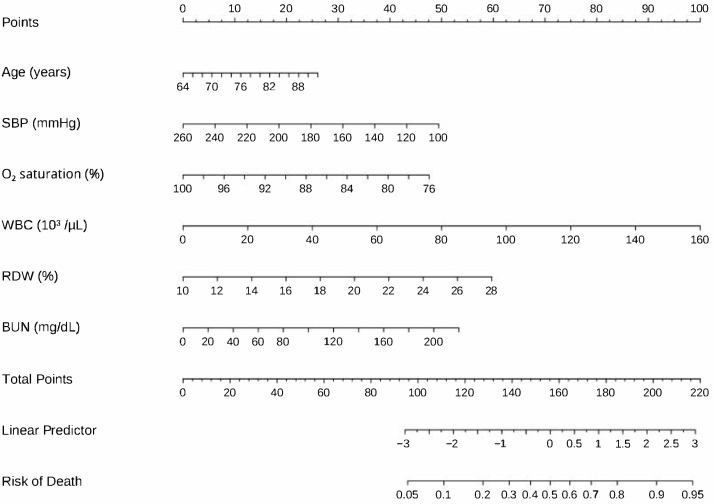
Predictive nomogram for calculating in-hospital mortality risk. Individual predictor values are converted to points via the upper scoring scale, totaled together, and subsequently mapped to the lower probability scale to determine mortality likelihood.

Utilizing the LASSO regression model with 10-fold cross-validation, we evaluated demographics, comorbidities, triage vitals, and laboratory measurements obtained at the initial ED visit. At the λ that minimized binomial deviance, eight variables retained non-zero coefficients: age, SBP, oxygen saturation, hypertension, congestive heart failure, WBC, RDW, and BUN (Figure S2). In the univariable regression, eight candidate predictors showed significant associations with in-hospital mortality: age (OR 1.47 per 10-year increase, 95% CI 1.08–2.01, *p* = 0.015), SBP (OR 1.24 per 10 mm Hg decrease, 95% CI 1.10–1.41, *p* = 0.001), oxygen saturation (OR 1.75 per 5-percentage-point decrease, 95% CI 1.16–2.56, *p* = 0.005), hypertension (OR 1.90, 95% CI 1.12–3.37, *p* = 0.021), congestive heart failure (OR 2.29, 95% CI 1.41–3.71, *p* = 0.001), WBC (OR 1.03 per 1 × 10^3^ µL^−1^ increase, 95% CI 1.01–1.06, *p* = 0.007), RDW (OR 1.20 per 1% increase, 95% CI 1.10–1.31, *p* < 0.001), and BUN (OR 1.02 per 1 mg dL^−1^ increase, 95% CI 1.01–1.02, *p* < 0.001) (Table 2). When these eight variables were entered into a multivariable logistic regression, six remained independently significant: age (OR 1.62, 95% CI 1.17–2.27, *p* = 0.004), SBP (OR 1.16, 95% CI 1.03–1.32, *p* = 0.020), oxygen saturation (OR 1.65, 95% CI 1.08–2.44, *p* = 0.015), WBC (OR 1.03, 95% CI 1.01–1.06, *p* = 0.008), RDW (OR 1.15, 95% CI 1.03–1.26, *p* = 0.008), and BUN (OR 1.01, 95% CI 1.00–1.02, *p* = 0.013). A nomogram was subsequently constructed from these six independently significant predictors to facilitate bedside estimation of an individual patient’s probability of in-hospital mortality (Figure 2).

### 3.4. Model Performance and Bootstrap Internal Validation

**Figure 3:**
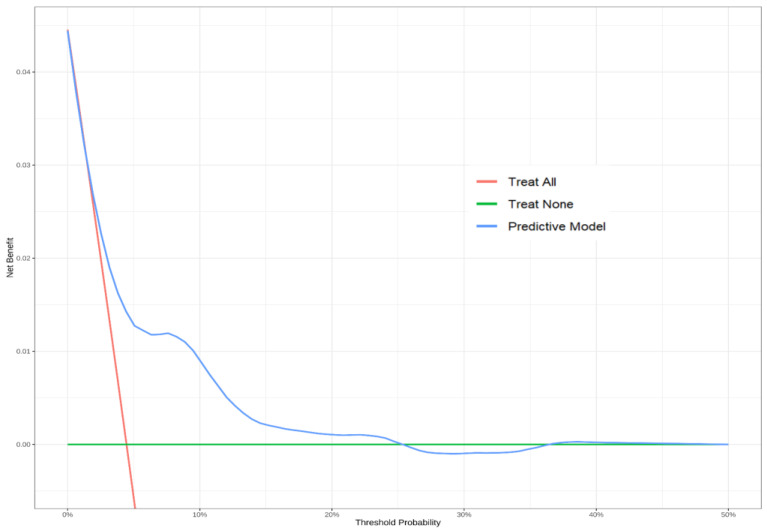
Decision curve analysis. The graph features the predictive model as the blue curve. The *y*-axis denotes net benefit, and the *x*-axis represents the threshold probability. The red and green lines indicate the clinical benefits for universal treatment and no treatment scenarios, respectively. The net clinical benefit provided by algorithms is shown by the vertical distance between the curves at different risk thresholds.

**Figure 4:**
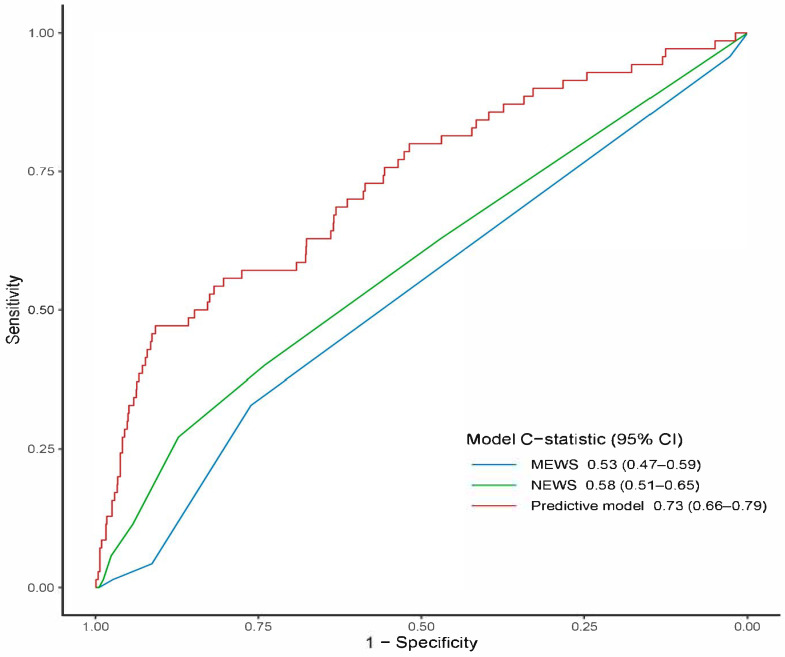
Receiver operating characteristic curves of the predictive model for in-hospital mortality compared with NEWS and MEWS. NEWS: national early warning score; MEWS: modified early warning score.

The resulting six-variable model achieved an apparent C-statistic of 0.73 (95% CI 0.66–0.79). After 1000-sample bootstrap validation, optimism was 0.02, yielding a bias-corrected C-statistic of 0.71. Bootstrap-derived calibration analysis using 1000 resampled iterations revealed close concordance between predicted mortality probabilities and actual observed outcomes (Hosmer–Lemeshow χ^2^  *p* = 0.222) (Figure S3). DCA demonstrated superior net clinical benefit across a wide threshold-probability range (5–25%) relative to default “treat-all” or “treat-none” strategies (Figure 3). Discrimination significantly outperformed the MEWS (C-statistic 0.53; 95% CI 0.47–0.59) and NEWS (C-statistic 0.58; 95% CI 0.51–0.65); DeLong’s test *p* < 0.001 for both pairwise comparisons (Figure 4).

## 4. Discussion

In this retrospective study of 1571 culture-confirmed, hemodynamically stable older adults admitted with UTI, six routine ED variables—age, lower SBP, lower oxygen saturation, higher WBC, elevated RDW, and higher BUN—were independently associated with in-hospital mortality. A nomogram built on these predictors was well calibrated and achieved moderate discrimination (C-statistic 0.73), outperforming MEWS (0.53) and NEWS (0.58), and thus offers potential to refine ED triage and management in this older population. To our knowledge, this is the first study and predictive model derived specifically for hemodynamically stable older ED adults with UTI.

Recent nomograms aimed at predicting adverse outcomes in UTI or urosepsis have generally reported slightly higher discrimination than our predictive nomogram, but they have relied on markedly more complex inputs and settings that limit bedside adoption. Wei et al. built an ICU-based model for elderly urosepsis that combined the Glasgow Coma Scale (GCS), RDW, WBC, and the need for invasive ventilation, reaching an internal C-statistic of 0.82, yet the inclusion of organ-support variables available only after admission constrains early triage use [28]. Zhang et al. analyzed 6551 mixed-age ICU encounters and produced an 11-item nomogram incorporating Acute Physiology Score III (APS-III), multiple comorbidities and detailed differential counts; their validation C-statistic was 0.83, but the requirement for a full severity-of-illness score and laboratory differentials limits practicality in overcrowded Eds [29]. Even procedure-specific tools—such as Hu’s nomogram for urosepsis after ureteral calculi, which achieved an external C-statistic of 0.76 with seven peri-operative factors—target narrow surgical subgroups rather than the broad spectrum of community-onset infection we studied [30]. By contrast, our model is derived entirely from the first ED set of vitals and basic laboratories and is restricted to hemodynamically stable adults ≥ 65 years—an under-represented yet high-risk cohort. Although the simpler six-variable profile yields slightly lower discrimination, its parsimony and immediate availability argue for greater real-world usability in front-door decision-making. Traditional early warning scores were conceived to flag general in-hospital deterioration rather than infection-specific risk. Consequently, their prognostic power erodes in older adults with UTI. A meta-analysis of NEWS in >100,000 patients reported a pooled C-statistic of 0.70, but the elder subgroup fell to 0.63 with sensitivities near 0.55 [31]. Similar reviews of MEWS show discrimination rarely exceeding 0.65 for sepsis-related mortality in seniors [32].

The variables anchoring our nomogram align with well-established mortality determinants. Hypoxemia captured by oxygen saturation likely signals impaired gas exchange, micro-circulatory shunting, or occult pulmonary infection; pre-hospital data show that each 3-point fall in oxygen saturation below 95% reduces 30-day survival in sepsis, independent of age and comorbidity [33,34]. Similarly, modest hypotension within the ostensibly “stable” range reflects loss of cardiovascular reserve; in one study of 833 ED patients ≥ 65 years with suspected infection, SBP ≤ 100 mmHg quadrupled adjusted in-hospital mortality versus SBP ≥ 140 mmHg, with a near-linear gradient across intermediate categories [35]. This study suggested that the commonly used threshold for hypotension (SBP below 90–100 mmHg) may not be clinically meaningful for the risk stratification of older ED patients with suspected infection. These findings support using continuous SBP and oxygen saturation values—rather than binary thresholds—to detect elders at hidden risk. RDW contributes a hematologic dimension. Inflammation and oxidative stress inhibit erythrocyte maturation and promote anisocytosis; across 16,423 ICU sepsis admissions, RDW > 15% conferred a two-fold adjusted odds of death, and the effect was even stronger in patients over 70 years [36]. An ED study focused on frail elders corroborated this relationship, reporting a stepwise rise in 30-day mortality across RDW quartiles [37]. Because RDW is part of every complete blood count, its inclusion adds discriminatory power at zero extra cost. Finally, elevated BUN integrates pre-renal azotemia, catabolism, and intrinsic renal dysfunction, all common in geriatric UTIs where dehydration and cytokine-mediated renal injury intersect. A multi-center study of elderly genitourinary infections found that each 5 mg dL^−1^ rise in BUN increased adjusted in-hospital mortality by 11% and improved risk reclassification beyond vital signs alone [38]. Together, these variables capture complementary circulatory, respiratory, hematologic, and renal stresses that often precede overt deterioration, providing a biologically sound foundation for our ED-based, elder-specific predictive model.

Our study offers some methodological advantages that bolster confidence in the nomogram’s validity. The cohort of 1571 culture-confirmed UTIs represents one of the largest single-center elder-specific ED datasets to date. All modeling steps were repeated during 1000-sample bootstrap validation, a method widely endorsed for the internal validation of prediction models [39]. Furthermore, we combined modern LASSO shrinkage with an interpretable logistic framework, merging parsimony with bedside transparency, and benchmarked performance directly against MEWS and NEWS—comparators rarely included in prior UTI prognostic studies. Clinically, the six-variable tool can meaningfully refine front-door decision-making. Because every predictor is readily available on ED arrival, the nomogram could be embedded as an auto-populated module in the electronic health record. A real-time display of predicted mortality would allow clinicians to (i) allocate high-acuity beds to seemingly “stable” elders whose risk exceeds a prespecified threshold, (ii) trigger early sepsis bundles or ICU consultation, and (iii) guide conversations about prognosis and goals of care with patients and families, an increasingly important component of geriatric emergency medicine [40,41]. Finally, the nomogram provides a standardized baseline risk measure that can adjust for case-mix in quality-improvement audits and antibiotic-stewardship trials, where outcome differences must be disentangled from underlying severity.

### Limitations

This study has several limitations. First, it derives from a single urban, tertiary-care ED in the United States; practice patterns, case mix and microbiology may differ elsewhere, so external validity is uncertain. Second, the retrospective design relies on routinely collected data and is therefore subject to residual confounding and documentation bias. Third, although only 70 in-hospital deaths occurred, the model includes six predictors, giving an events-per-variable ratio of approximately 11.7—above the conventional ≥ 10 threshold recommended for logistic regression—yet coefficient stability and C-statistic precision should still be interpreted with caution. Fourth, although internal optimism was addressed with bootstrap resampling, we did not perform geographic or temporal external validation, which is essential before clinical implementation. Fifth, frailty and functional-status measures—key prognostic factors in geriatric infection—were unavailable in MIMIC-IV-ED, as were clinician gestalt variables such as symptom duration or nursing home residence. Sixth, UTI diagnoses and comorbidities were identified through ICD-9/-10 codes; even with validated algorithms, misclassification is possible and could dilute true associations. Additionally, although restricting the derivation cohort to culture-confirmed UTIs reduced diagnostic noise, it may also introduce spectrum bias. In routine ED practice, 30–45% of older adults who start antibiotics for suspected UTI later prove culture-negative, often because of non-infectious mimics or prior antimicrobial exposure. Our nomogram uses only triage vital signs and first laboratory values—information available long before culture results—so it can be calculated in real time; nonetheless, its accuracy in culture-negative or clinically diagnosed cases remains untested. Emerging culture-independent diagnostics could help bridge this gap. A recent systematic review of the urobiome reports that metagenomic next-generation sequencing yields approximately 81% sensitivity and 92% specificity for pathogen detection in urine—results delivered within hours—and highlights a broader shift toward rapid, comprehensive ED testing [42]. Future studies should therefore validate the nomogram in all patients treated empirically for UTI and examine whether pairing the nomogram with these rapid assays improves calibration and clinical utility. Finally, GCS readings were not recorded in this ED dataset; interpreting absent neurological parameters as “normal” is the recommended workaround for MEWS and NEWS but may undermine their predictive reliability, limiting fair comparison with our tailored model. Because our model was derived entirely from a single U.S. tertiary-care ED, it should be considered hypothesis-generating until validated in external, multicenter datasets. Prospective multicenter studies that incorporate frailty indices, neurological assessments, and contemporary treatment protocols are therefore required to externally validate the nomogram and to recalibrate decision thresholds for diverse healthcare environments.

## 5. Conclusions

In this large cohort of hemodynamically stable older adults with UTI, we developed a six-variable ED nomogram—age, SBP, oxygen saturation, WBC, RDW, and BUN—that outperformed generic early warning scores and offers rapid, bedside estimation of in-hospital mortality. Its predictors are routinely available on ED arrival, facilitating earlier ICU consultation, informed goals-of-care discussions and risk-adjusted quality initiatives. Because the model was developed in a single U.S. tertiary-care ED, it remains hypothesis-generating until validated in external, multicenter cohorts. Prospective multicenter studies should confirm its generalizability and determine whether nomogram-guided care improves patient outcomes.

## Figures and Tables

**Table 1:** Comparison of variables between patients with and without in-hospital mortality.

Variables	Total (n = 1571)	Non-Survivors (n = 70)	Survivors (n = 1501)	*p* Value
Age (years), median [IQR]	79.0 [72.0–85.0]	82.0 [74.0–88.0]	79.0 [72.0–85.0]	0.014 *
Sex (male), n (%)	524 (33.4)	25 (35.7)	499 (33.2)	0.765
Race, n (%)				0.504
Asian	42 (2.7)	0 (0.0)	42 (2.8)	
Black	201 (12.8)	10 (14.3)	191 (12.7)	
Hispanic	64 (4.1)	3 (4.3)	61 (4.1)	
Other	91 (5.8)	2 (2.9)	89 (5.9)	
White	1173 (74.7)	55 (78.6)	1118 (74.5)	
Insurance, n (%)				0.489
Medicaid	23 (1.5)	2 (2.9)	21 (1.4)	
Medicare	1125 (71.6)	47 (67.1)	1078 (71.8)	
Other	423 (26.9)	21 (30.0)	402 (26.8)	
Emergency Severity Index, n (%)				0.782
Level 1	136 (8.7)	8 (11.4)	128 (8.5)	
Level 2	824 (52.5)	37 (52.9)	787 (52.4)	
Level 3	605 (38.5)	25 (35.7)	580 (38.6)	
Level 4	6 (0.4)	0 (0.0)	6 (0.4)	
Vital signs at triage, median [IQR]				
body temperature (°C)	37.6 [37.2–37.8]	37.5 [37.2–37.7]	37.6 [37.3–37.8]	0.795
Systolic blood pressure (mmHg)	134.0 [120.0–152.0]	122.5 [115.0–138.0]	135.0 [121.0–152.0]	<0.001 *
Diastolic blood pressure (mmHg)	71.0 [61.0–81.0]	68.0 [61.0–78.0]	71.0 [61.0–81.0]	0.153
Heart rate (beats/min)	81.0 [70.0–91.5]	84.0 [72.0–95.0]	81.0 [70.0–91.0]	0.387
Respiratory rate (beats/min)	18.0 [16.0–19.0]	18.0 [16.2–20.0]	18.0 [16.0–19.0]	0.200
Oxygen saturation (%)	98.0 [96.0–99.0]	97.5 [95.0–99.0]	98.0 [96.0–99.0]	0.164
Comorbidities, n (%)				
Hypertension	957 (60.9)	52 (74.3)	905 (60.3)	0.026 *
Diabetes mellitus	490 (31.2)	29 (41.4)	461 (30.7)	0.078
Congestive heart failure	472 (30.0)	34 (48.6)	438 (29.2)	0.001 *
Prior stroke	221 (14.1)	7 (10.0)	214 (14.3)	0.409
Liver disease	104 (6.6)	7 (10.0)	97 (6.5)	0.359
Chronic kidney disease	471 (30.0)	26 (37.1)	445 (29.6)	0.228
Malignancy	221 (14.1)	15 (21.4)	206 (13.7)	0.102
Charlson comorbidity index	6.0 [4.0–9.0]	8.0 [4.0–11.0]	6.0 [4.0–9.0]	0.002 *
Laboratory data (serum), median [IQR]				
WBC (10^3^/uL)	8.4 [6.2–11.1]	9.9 [7.1–14.3]	8.2 [6.2–11.1]	0.002 *
Hemoglobin (g/dL)	10.6 [9.1–12.0]	9.9 [8.5–11.6]	10.6 [9.1–12.0]	0.052
RDW (%)	14.7 [13.6–16.3]	15.7 [14.1–18.0]	14.7 [13.6–16.2]	<0.001 *
Platelet count (10^3^/uL)	214.0 [162.0–278.0]	205.0 [153.5–262.0]	215.0 [163.0–278.0]	0.338
PT (s)	13.1 [11.8–17.3]	14.3 [12.4–16.4]	13.1 [11.8–17.6]	0.175
Sodium (mEq/L)	139.0 [136.0–142.0]	138.0 [135.0–142.8]	139.0 [136.0–142.0]	0.304
Potassium (mEq/L)	4.1 [3.8–4.5]	4.2 [3.8–4.6]	4.1 [3.8–4.5]	0.522
AST (U/L)	24.5 [18.0–37.0]	30.0 [21.0–42.0]	24.0 [18.0–37.0]	0.073
ALT (U/L)	18.0 [12.0–29.0]	19.5 [13.0–34.5]	18.0 [12.0–28.0]	0.130
Total bilirubin (mg/dL)	0.5 [0.3–0.8]	0.5 [0.4–1.0]	0.5 [0.3–0.7]	0.054
Glucose (mg/dL)	110.0 [93.0–140.0]	111.0 [94.0–143.0]	110.0 [93.0–140.0]	0.906
BUN (mg/dL)	23.0 [16.0–35.2]	31.0 [20.2–44.5]	23.0 [16.0–35.0]	0.001 *
Creatinine (mg/dL)	1.0 [0.8–1.6]	1.3 [0.8–1.7]	1.0 [0.8–1.5]	0.048 *
Urinalysis, median [IQR]				
WBC (/HPF)	20.0 [6.0–57.0]	16.5 [3.8–57.8]	20.0 [6.0–57.0]	0.406
RBC (/HPF)	4.0 [2.0–14.0]	6.0 [1.2–13.5]	4.0 [2.0–14.0]	0.992
Urine culture, n (%)				<0.001 *
*Escherichia coli*	583 (37.1%)	15 (21.4%)	568 (37.8%)	
*Enterococcus* sp.	247 (15.7%)	10 (14.3%)	237 (15.8%)	
Yeast	135 (8.6%)	17 (24.3%)	118 (7.9%)	
*Klebsiella* sp.	163 (10.4%)	8 (11.4%)	155 (10.3%)	
*Proteus* sp.	81 (5.2%)	4 (5.7%)	77 (5.1%)	
*Pseudomonas* sp.	77 (4.9%)	4 (5.7%)	73 (4.9%)	
Other microorganisms	285 (18.1%)	12 (17.1%)	273 (18.2%)	
Early warning score, median [IQR]				
MEWS	1.0 [1.0–1.0]	1.0 [1.0–2.0]	1.0 [1.0–1.0]	0.296
NEWS	1.0 [0.0–2.0]	1.0 [0.0–3.0]	1.0 [0.0–2.0]	0.012 *
Outcome, n (%)				
ICU admission	227 (14.4)	52 (74.3)	175 (11.7)	<0.001 *

IQR: interquartile range; WBC: white blood cell; BUN: blood urea nitrogen; RDW: red cell distribution width; PT: prothrombin time; AST: aspartate aminotransferase; ALT: alanine aminotransferase; RBC: red blood cell; ICU: intensive care unit; MEWS: modified early warning score; NEWS: national early warning score. * *p* value < 0.05

**Table 2:** Univariable and multivariable logistic LASSO regression analysis of risk factors for in-hospital mortality.

Variables	Univariable		Multivariable	
	OR (95% CI)	*p* Value	OR (95% CI)	*p* Value
Age (per 10-year increase)	1.47 (1.08–2.01)	0.015 *	1.62 (1.17–2.27)	0.004 *
Systolic blood pressure (per 10 mm Hg decrease)	1.24 (1.10–1.41)	0.001 *	1.16 (1.03–1.32)	0.020 *
Oxygen saturation (per 5 percentage-point decrease)	1.75 (1.16–2.56)	0.005 *	1.65 (1.08–2.44)	0.015 *
Hypertension (yes vs. no)	1.90 (1.12–3.37)	0.021 *	1.43 (0.75–2.77)	0.284
Congestive heart failure (yes vs. no)	2.29 (1.41–3.71)	0.001 *	1.36 (0.75–2.49)	0.315
WBC (per 1 × 10^3^ µL^−1^ increase)	1.03 (1.01–1.06)	0.007 *	1.03 (1.01–1.06)	0.008 *
RDW (per 1 percentage-point increase)	1.20 (1.10–1.31)	<0.001 *	1.15 (1.03–1.26)	0.008 *
BUN (per 1 mg dL^−1^ increase)	1.02 (1.01–1.02)	<0.001 *	1.01 (1.00–1.02)	0.013 *

OR: odds ratio; 95% CI: 95% confidence interval; WBC: white blood cell; BUN: blood urea nitrogen; RDW: red cell distribution width. * *p* value < 0.05.

## Data Availability

Our research utilized publicly available data housed within the MIMIC-IV-ED database https://physionet.org/content/mimic-iv-ed/2.2/ (accessed on 1 January 2025). Access protocols incorporate controlled sharing mechanisms to protect patient confidentiality despite de-identification processes. Investigators must navigate a multi-step approval pathway encompassing the following: credentialed registration with the PhysioNet platform (https://physionet.org/, 1 January 2025), successful completion of data stewardship training requirements, and formal acceptance of binding data use agreements governing research applications.

## Supplementary Materials

The following supporting information can be downloaded at: https://www.mdpi.com/article/10.3390/medicina61091625/s1, Figure S1: Missing values for variables in the entire cohort; Figure S2: Tuning parameter (λ) selection in the LASSO model using 10-fold cross-validation via minimum deviance; Figure S3: Calibration curve for the predictive nomogram. The x-axis depicts the predicted mortality risk, while the y-axis shows the actual risk of mortality. The diagonal dotted line illustrates a perfect prediction from an ideal model. The short-dashed line represents the nomogram’s apparent prediction, with the solid line indicating the nomogram’s performance after bias-correction through bootstrapping (B = 1000 repetitions).
