# Supplementary material for: Risk Factors and Development of a Predictive Model for In-Hospital Mortality in Hemodynamically Stable Older Adults with Urinary Tract Infection
Source: Medicina (Kaunas). 2025 Sep 8;61(9):1625. doi: 10.3390/medicina61091625 (PMC12471474; doi:10.3390/medicina61091625)

Figure S1. Missing values for variables in the entire cohort

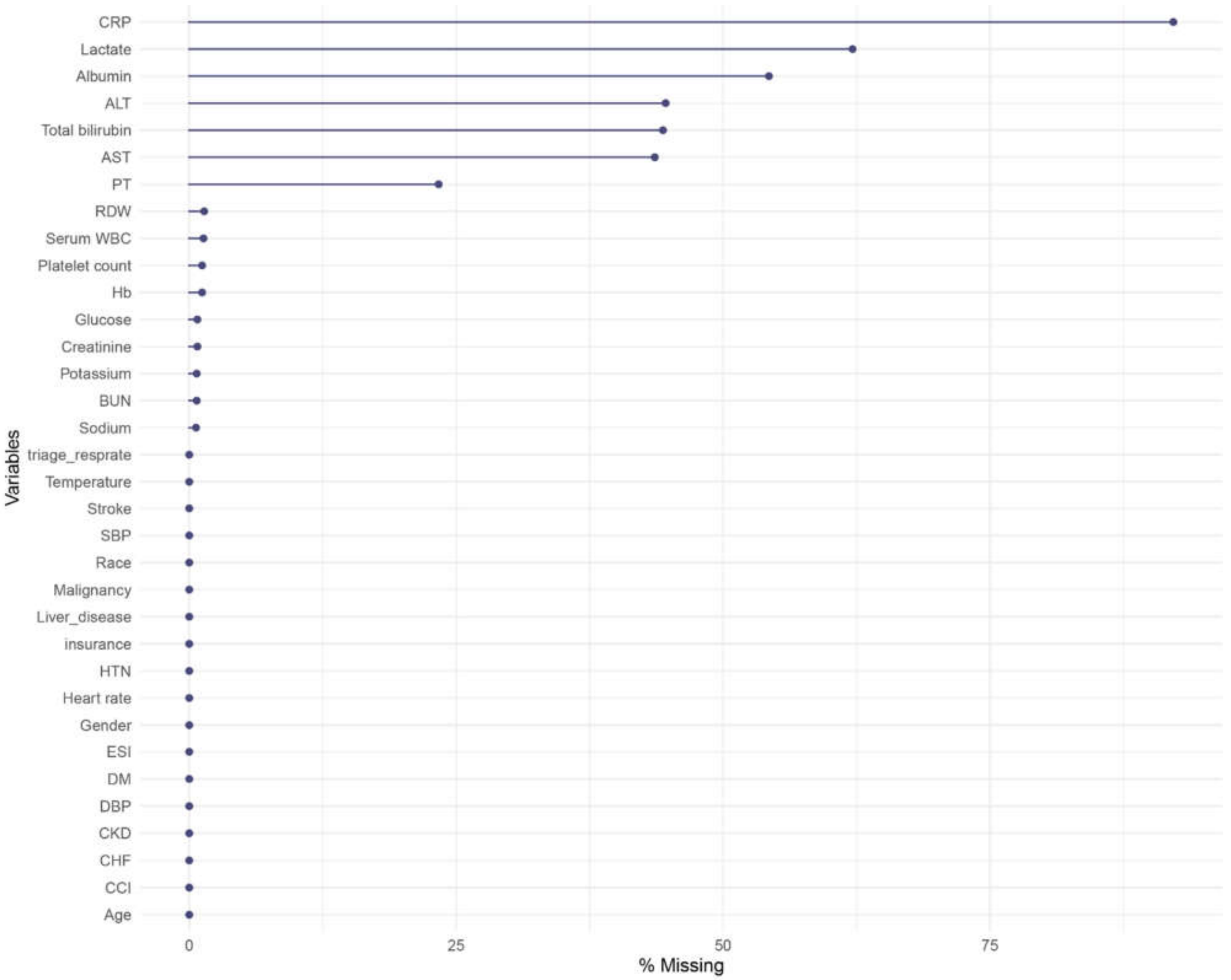

Figure S2. Tuning parameter (λ) selection in the LASSO model using 10-fold cross-validation via minimum deviance.

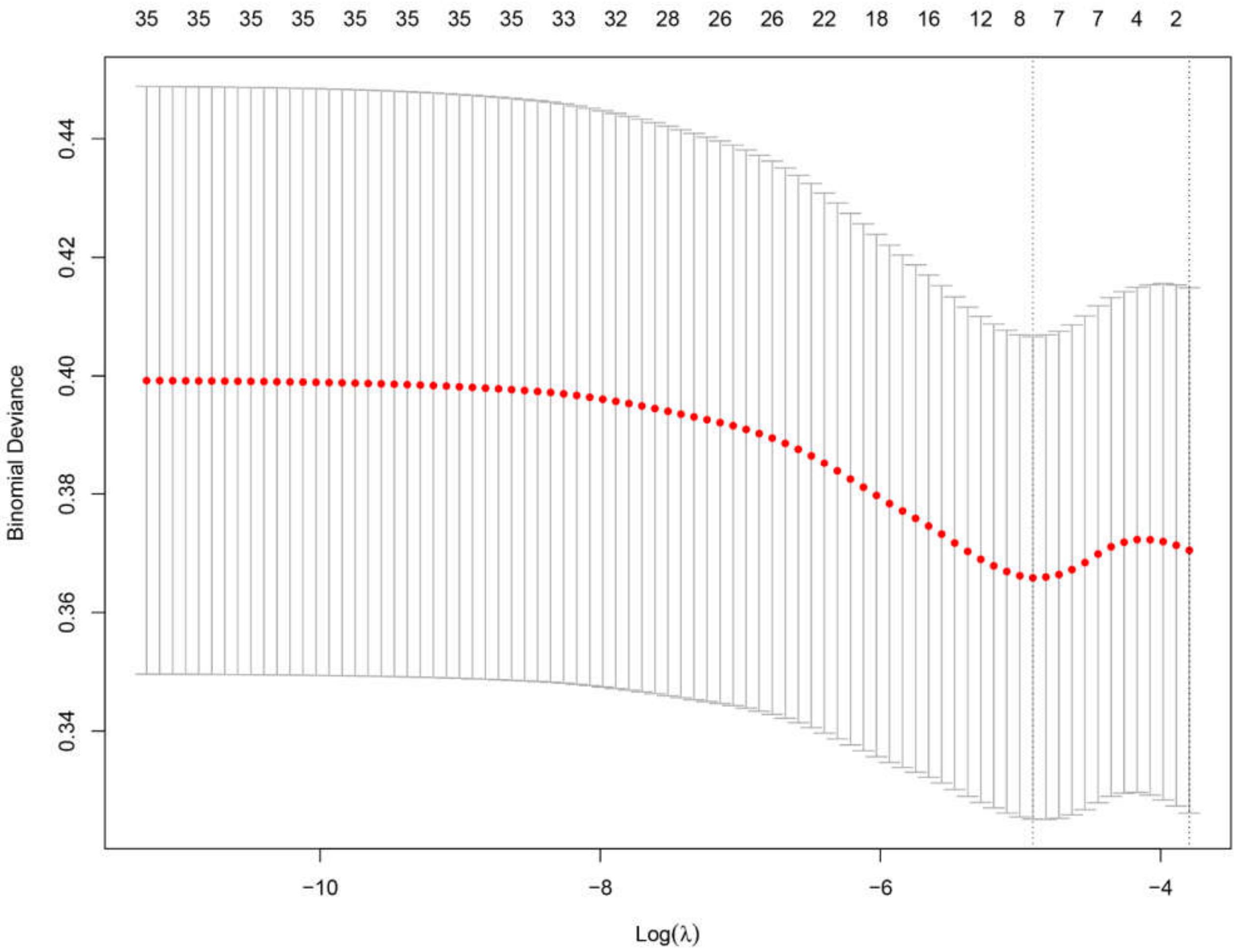

Figure S3. Calibration curve for the predictive nomogram. The x-axis depicts the predicted mortality risk, while the y-axis shows the actual risk of mortality. The diagonal dotted line illustrates a perfect prediction from an ideal model. The short-dashed line represents the nomogram's apparent prediction, with the solid line indicating the nomogram's performance after bias-correction through bootstrapping (B = 1000 repetitions).

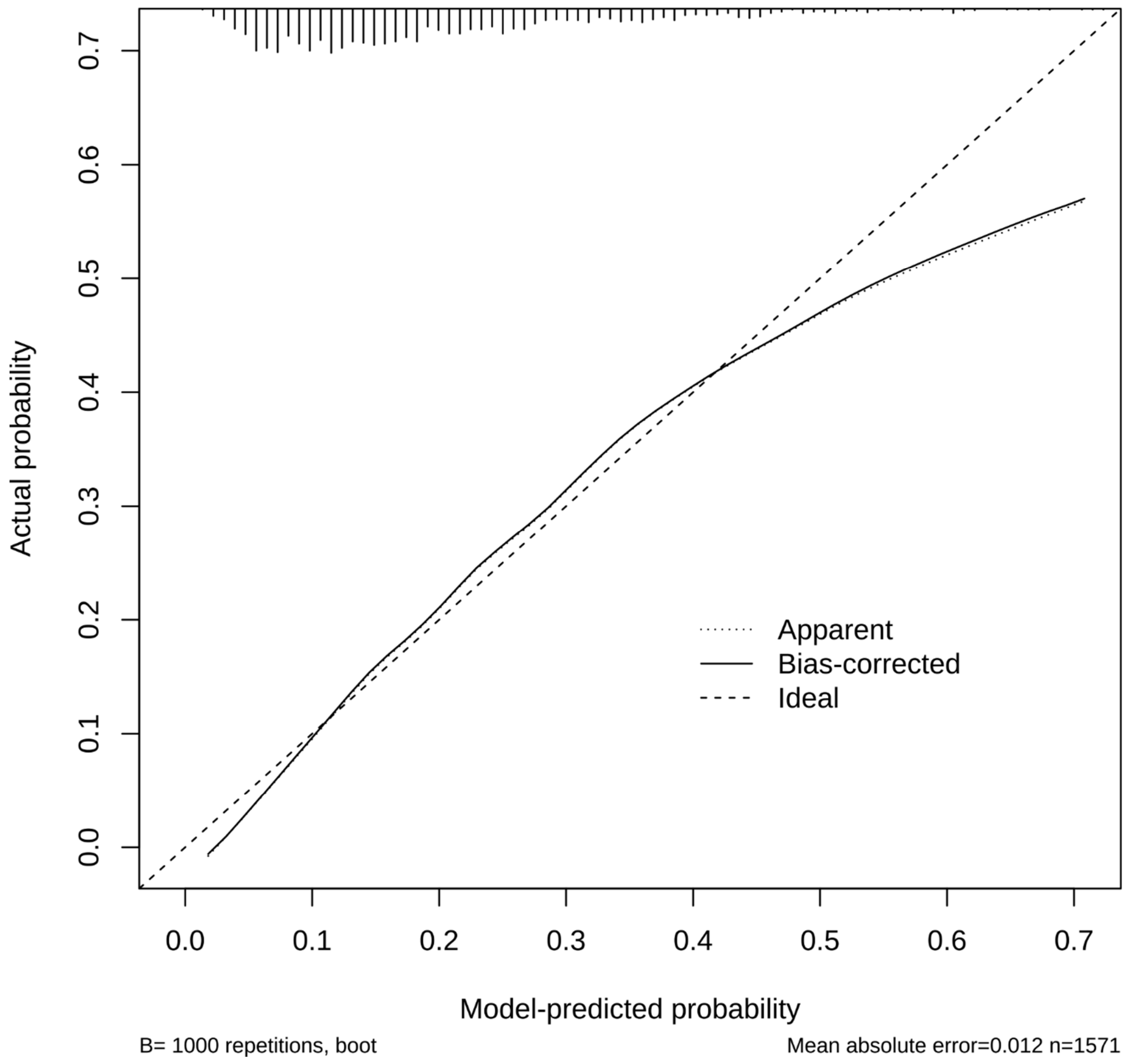

Supplement: Supplementary file 1 [file medicina-61-01625-s001.zip › Supplementary materials v2.pdf]
